# Diversity and Distribution of Sulfur Oxidation-Related Genes in *Thioalkalivibrio*, a Genus of Chemolithoautotrophic and Haloalkaliphilic Sulfur-Oxidizing Bacteria

**DOI:** 10.3389/fmicb.2019.00160

**Published:** 2019-02-14

**Authors:** Tom Berben, Lex Overmars, Dimitry Y. Sorokin, Gerard Muyzer

**Affiliations:** ^1^Microbial Systems Ecology, Department of Freshwater and Marine Ecology, Institute for Biodiversity and Ecosystem Dynamics, University of Amsterdam, Amsterdam, Netherlands; ^2^Winogradsky Institute for Microbiology, Research Center of Biotechnology, Russian Academy of Sciences, Moscow, Russia; ^3^Department of Biotechnology, Delft University of Technology, Delft, Netherlands

**Keywords:** *Thioalkalivibrio*, soda lakes, chemolithoautotrophs, sulfur oxidation, thiocyanate oxidation, denitrification

## Abstract

Soda lakes are saline alkaline lakes characterized by high concentrations of sodium carbonate/bicarbonate which lead to a stable elevated pH (>9), and moderate to extremely high salinity. Despite this combination of extreme conditions, biodiversity in soda lakes is high, and the presence of diverse microbial communities provides a driving force for highly active biogeochemical cycles. The sulfur cycle is one of the most important of these and bacterial sulfur oxidation is dominated by members of the obligately chemolithoautotrophic genus *Thioalkalivibrio*. Currently, 10 species have been described in this genus, but over one hundred isolates have been obtained from soda lake samples. The genomes of 75 strains were sequenced and annotated previously, and used in this study to provide a comprehensive picture of the diversity and distribution of genes related to dissimilatory sulfur metabolism in *Thioalkalivibrio*. Initially, all annotated genes in 75 *Thioalkalivibrio* genomes were placed in ortholog groups and filtered by bi-directional best BLAST analysis. Investigation of the ortholog groups containing genes related to sulfur oxidation showed that flavocytochrome *c (fcc)*, the truncated *sox* system, and sulfite:quinone oxidoreductase (*soe*) are present in all strains, whereas dissimilatory sulfite reductase (*dsr*; which catalyzes the oxidation of elemental sulfur) was found in only six strains. The heterodisulfide reductase system (*hdr*), which is proposed to oxidize sulfur to sulfite in strains lacking both *dsr* and *soxCD*, was detected in 73 genomes. Hierarchical clustering of strains based on sulfur gene repertoire correlated closely with previous phylogenomic analysis. The phylogenetic analysis of several sulfur oxidation genes showed a complex evolutionary history. All in all, this study presents a comprehensive investigation of sulfur metabolism-related genes in cultivated *Thioalkalivibrio* strains and provides several avenues for future research.

## Introduction

The biogeochemical sulfur cycle is a complex network of biotic and abiotic reactions, involving both organic and inorganic sulfur compounds. This is due to the many possible oxidation states of the sulfur atom, from −II (sulfide) to +VI (sulfate) allowing for multiple energy-generating red-ox transformations in both oxidative and reductive directions. Sulfur cycling is linked to other biogeochemical redox cycles including those of carbon, nitrogen and metals (through interactions with sulfides), and is an important driver of microbial growth and element conversions in a large number of different environments (Sorokin et al., 2011; Bowles et al., 2014; Wasmund et al., 2017). Sulfur-oxidizing bacteria (SOB) derive energy from the oxidation of reduced S compounds, whereas sulfidogens (sulfate-, sulfite-, sulfur- and thiosulfate-reducing, as well as sulfur-disproportionating prokaryotes) utilize oxidized sulfur compounds as electron acceptors in the absence of oxygen. These organisms are the main driving force of the microbial dissimilatory sulfur cycle. Examples of environments with the highest sulfur cycling activity are marine sediments (Wasmund et al., 2017), deep-sea “Black Smoker” hydrothermal vents (Jannasch and Mottl, 1985), acid mine drainage sites (Méndez-García et al., 2015) and alkaline soda lakes (Sorokin et al., 2015). The latter are of particular interest to our understanding of microbial sulfur metabolism, because of some unique features of sulfur chemistry at high pH, such as the reduced toxicity of hydrosulfide (HS^−^) and the stability of polysulfide (*S*_n_^2^) (Sorokin et al., 2011).

Soda lakes are double extreme environments characterized by a stable, elevated pH (>pH 9) caused by the presence of high concentrations of sodium carbonate/bicarbonate which can reach up to saturation and result in molar concentrations of soluble alkalinity. They can be found around the globe in arid regions and are formed by evaporation of CO_2_-rich groundwaters in the absence of divalent cations, such as Ca^2+^ and Mg^2+^ (Sorokin et al., 2014). Despite the challenges posed by these saline alkaline conditions, soda lakes support a diverse microbial community, which drives active carbon, nitrogen and sulfur cycles (Sorokin et al., 2014, 2015). Samples taken from south Siberian soda lakes showed high rates of sulfidogenesis (Foti et al., 2007; Sorokin et al., 2010); however, to our knowledge, the rates of sulfide oxidation in soda lakes have not been measured. These rates are likely similarly high, given the fact that sulfide is absent in the top 1–2 cm sediment layer of the soda lakes (Sorokin et al., 2010). Sulfide and other reduced sulfur compounds are oxidized by a community consisting of both phototrophic and chemotrophic SOB (Sorokin et al., 2014). The gammaproteobacterial genus *Thioalkalivibrio* (Sorokin et al., 2013) is the most abundant group of sulfur-oxidizing bacteria in soda lakes, which was recently confirmed by metagenomic analysis (Vavourakis et al., 2018). *Thioalkalivibrio* is one of the best-studied groups of bacteria from soda lakes, with nearly a hundred isolates originating from hypersaline soda lakes in seven regions of the world: the Kulunda Steppe, the Buriatia region, the Transbaikal region and North-Eastern Mongolia in Central Asia, Wadi al Natrun (Libyan desert) in Egypt, the East-African Rift Valley in Kenya, and California and Washington State in North-America. Furthermore, *Thioalkalivibrio* species are the dominant SOB in so-called Thiopaq systems, full-scale bioreactor installations used to remove sulfide from different gas streams at haloalkaline conditions (Sorokin et al., 2008, 2012; Janssen et al., 2009).

Currently, the genus *Thioalkalivibrio* contains 10 validly described species: *Tv. versutus* (type species), *Tv. nitratis*, *Tv. denitrificans* (Sorokin et al., 2001a), *Tv. paradoxus*, *Tv. thiocyanoxidans* (Sorokin et al., 2002b), *Tv. jannaschii* (Sorokin et al., 2002a)*, Tv. thiocyanodenitrificans* (Sorokin et al., 2004), *Tv. nitratireducens* (Sorokin, 2003), *Tv. halophilus* (Banciu et al., 2004), and *Tv. sulfidiphilus* (Sorokin et al., 2012). These 10 species show a high degree of metabolic flexibility: every isolate from all strains can oxidize sulfide, thiosulfate, elemental sulfur, and polysulfide. Some strains can additionally oxidize tetrathionate, carbon disulfide, and thiocyanate. Although most strains are obligate aerobes, three species are capable of anaerobic growth with either nitrate, nitrite, or nitrous oxide as the electron acceptor (*Tv. denitrificans*, *Tv. thiocyanodenitrificans*, and *Tv. nitratireducens*). Most of the isolates are capable of growth in saturated soda brines, with a total sodium concentration of over 4.3 M Na^+^ and pH up to 10.5.

Since whole genome sequencing has become widespread, our knowledge of metabolic potential has greatly increased, allowing microbiologists to conduct large-scale studies of the metabolic repertoire of multiple species. In the case of *Thioalkalivibrio*, the genomes of approximately 70 strains were sequenced as part of the Joint Genome Institute’s Community Science Program around 2012, and several additional type strains were sequenced independently. This created the largest dataset of genomes so far for any group of obligately lithoautotrophic bacteria. Only a small number of those genomes has been annotated and analyzed to date: (i) *Tv. sulfidiphilus* was selected for its relevance in sulfide-removing bioreactors (Muyzer et al., 2011a), (ii) *Thioalkalivibrio* sp. K90mix (Muyzer et al., 2011b) was selected for its extreme potassium tolerance, and (iii) *Tv. paradoxus*, *Tv. thiocyanoxidans*, and *Tv. thiocyanodenitrificans* (Berben et al., 2017) were selected for their ability to grow with thiocyanate (NCS^−^) as the sole electron donor. Additionally, a recent study used the complete set of genomic data to analyze the genomic diversity and phylogeny of the *Thioalkalivibrio* genus using modern *in silico* methods—such as average nucleotide identity (ANI), multi-locus sequence analysis (MLSA) and digital DNA–DNA hybridization (dDDH)—compared to classic DNA–DNA hybridization and 16S rRNA phylogeny (Ahn et al., 2017).

Although several studies have reported on individual aspects of sulfur metabolism in members of *Thioalkalivibrio*, to our knowledge there has been no genus-wide comprehensive analysis of sulfur oxidation-related genes present in the genomes. A complete understanding of the sulfur metabolism pathways in *Thioalkalivibrio* would provide insights into key microorganisms that carry out an important biogeochemical process in soda lakes and also may help to get insights and possibilities for process optimization of their application in desulfurization of bio- and industrial gas streams.

Here, we present the first comprehensive analysis of the distribution of genes encoding enzymes that catalyze the oxidation of reduced sulfur compounds across the genus *Thioalkalivibrio* and the diversity of metabolic strategies for dissimilatory sulfur metabolism in these bacteria. We mined the genomes of 75 strains for genes known to be involved in sulfur oxidation using ortholog prediction. These results were further refined with sequence and phylogenetic analyses. Our aim was to provide a comprehensive overview of the potential for the oxidation of a diverse set of sulfur compounds by members of the haloalkaliphilic genus *Thioalkalivibrio*.

## Materials and Methods

### *Thioalkalivibrio* Strains Studied

Cultures of 75 *Thioalkalivibrio* strains were obtained from the personal culture collection of D. Sorokin for genome sequencing. The enrichment and isolation of these strains were described previously (Sorokin et al., 2001a, 2002b, 2004, 2012; Sorokin, 2003; Banciu et al., 2004). A total of 72 of these strains were sequenced by the Joint Genome Institute (JGI) as part of the Community Science Program; cultivation, DNA extraction, sequencing, assembly and annotation procedures have been described previously (Muyzer et al., 2011a). The remaining strains (*Tv. versutus* AL2^T^, *Tv. denitrificans* ALJD^T^, and *Tv. halophilus* HL17^T^) were later sequenced as a separate project (Ahn et al., 2017), because the extracted DNA initially submitted to JGI was of insufficient quality for genome sequencing. Basic genome statistics and accession numbers are included in Supplementary Table S1.

### Ortholog Calling

All protein sequences of the 75 *Thioalkalivibrio* genomes listed in Supplementary Table S1 were subjected to an orthology prediction using OrthoMCL. First we used a locally installed NCBI blastp executable (version 2.7.1) with default parameters to perform all-vs.-all blast of protein sequences annotated in the *Thioalkalivibrio* genomes. OrthoMCL takes the output of the all-vs.-all blast and uses Markov clustering to calculate clusters of orthologous sequences. We utilized the default Markov inflation factor of 1.5, which balances clustering sensitivity and selectivity (Fischer et al., 2011). The output from OrthoMCL was parsed into a single gene presence/absence matrix using custom Python scripts. The ortholog group table is included as Supplementary Table S2. Ortholog groups representing sulfur oxidation genes were identified by blastp (Altschul et al., 1997), using sequences from well-studied organisms as queries. Sequences from *Allochromatium vinosum* were used for FccAB (Uniprot accessions: Q06529/Q06530), SoxAXYZB (Q1W3E4, D3RVS6, D3RVA1, D3RVA2, D3RVS5), DsrABC (O33998, D3RSN2, D3RSN6), AprBA (D3RSA0, D3RSA1), Sat (O66036) and SoeABC (NCBI accessions: ADC63403.1, ADC63402.1, ADC63401.1). For SQR a sequence from *Acidithiobacillus ferrooxidans* (B7JBP8) was used (a type I SQR), for SorAB sequences from *Starkeya novella* (Q9LA16, Q9LA15) and HdrABC (ADJ22511.1, ADJ22501.1, ADJ22509.1) from *Hyphomicrobium denitrificans*. For SOR, TcDH and ScnABC hydrolase we used sequences from *Thioalkalivibrio paradoxus* (WP_006748120.1), *Tv. thiocyanoxidans* (WP_019623383.1) and *Thiobacillus thioparus* (O66187, O66187, and O66188), respectively. We used a sequence similarity criterion of >30% and >50% query coverage, as well as organization of genes in operons in case of multi-enzyme systems (for example *soeABC*).

### Visualization of the Sulfur Oxidation Gene Distribution

**FIGURE 1:**
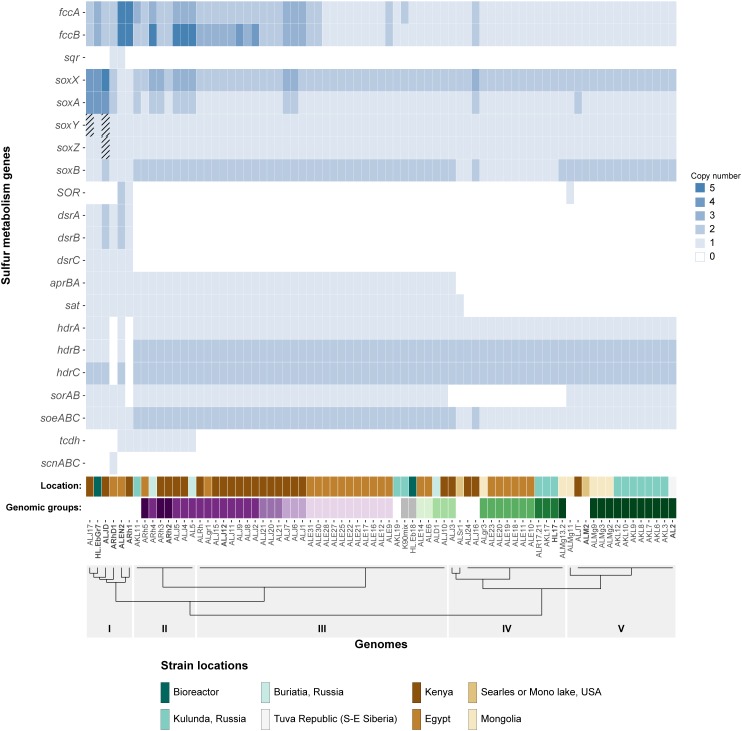
Heatmap showing the copy number of key sulfur oxidation genes in 75 *Thioalkalivibrio* strains. Cross-hatched cells represent genes which are likely present, but currently unconfirmed. Location denotes the geographical region from which the corresponding strain was isolated. The genomic groups colors correspond to species as defined by Ahn et al. (2017). Genomic groups comprising a single strain are not colored to maintain clarity. The dendrogram on the *x*-axis shows the Ward clustering of genomes based on presence/absence of the sulfur genes. Please note that *SOR* is sulfur oxygenase/reductase and *sorAB* is sulfite:cytochrome *c* oxidoreductase.

The matrix of genomes and gene copy numbers was visualized as a heatmap using ggplot2 in the R environment (Wickham, 2009). The dendrogram in Figure 1 was calculated by using Ward hierarchical clustering on the Euclidean distances on a presence/absence matrix of sulfur genes. This matrix contained all genes as separate rows, after clustering identical rows were collapsed for genes that are located in a single operon.

### Phylogenetic Analysis

Phylogenetic analysis of selected genes was performed as follows: all available sequences from *Thioalkalivibrio* (see Supplementary Table S1) were used. Additional sequences from both closely and more distantly related organisms were retrieved from the NCBI protein database, by using protein BLAST. Sequences were aligned using T-COFFEE (Di Tommaso et al., 2011) and the most likely amino acid substitution matrix was determined using prottest 3 (Abascal et al., 2005), which was the LG model (Le and Gascuel, 2008) with gamma-distributed rates and empirical frequencies (model parameter –m PROTGAMMALGF in RAxML) for all alignments. Maximum likelihood trees were calculated using RAxML 8.2.12 (Stamatakis, 2014, 2015), using the rapid bootstrap analysis algorithm. To test the reproducibility of tree branches either 250 or 500 bootstrap replicates were calculated for the final tree. Once generated, the tree was visualized in MEGA7 (Kumar et al., 2016), the root of the tree was placed on the outgroup branch and branches with sequences belonging to a single taxonomic group were collapsed for legibility.

## Results

An overview of sulfur oxidation-related genes identified in the 75 *Thioalkalivibrio* genomes is shown in Table 1. Genes for tetrathionate oxidation (tetrathionate hydrolase, tetrathionate:quinone oxidoreductase) were not detected in any of the *Thioalkalivibrio* genomes. A putative carbon disulfide hydrolase was only detected in the genome of *Tv. nitratireducens* ALEN 2^T^, including the characteristic ‘Phe–Phe’ motif (Smeulders et al., 2011).

**Table 1:** Overview of genes included in the analysis.

Abbreviation	Full name	Function in sulfur metabolism in *Thioalkalivibrio*	Reference
*fccAB*	Flavocytochrome *c* sulfide dehydrogenase	H_2_S/HS^−^ → S^0^	Chen et al., 1994
*sqr*	Sulfide:quinone reductase	H_2_S/HS^−^ → S^0^	Marcia et al., 2009
*soxABXYZ*		S_2_O_3_^2−^ → SO_4_^2−^ + S^0^	Kelly et al., 1997; Friedrich et al., 2000
*SOR*	Sulfur oxygenase/reductase	S^0^ → H_2_S + S_2_O_3_^2−^ + SO_3_^2−^	Rühl et al., 2017
*dsr*^∗^	(Reverse) dissimilatory sulfite reductase	S^0^ → SO_3_^2−^	Pott and Dahl, 1998; Dahl et al., 2005; Venceslau et al., 2014
*hdr*^†^	Heterodisulfide reductase system	S^0^ → SO_3_^2−^	Quatrini et al., 2009; Dahl, 2015; Boughanemi et al., 2016; Cao et al., 2018
*sorAB*	Sulfite:cytochrome *c* oxidoreductase	SO_3_^2−^ → SO_4_^2−^	Kappler et al., 2000
*soeABC*	Sulfite:quinone oxidoreductase	SO_3_^2−^ → SO_4_^2−^	Dahl et al., 2013
*aprBA*	APS reductase	SO_3_^2−^ → APS	Fritz et al., 2000
*sat*	Sulfate adenylyltransferase	APS → SO_4_^2−^	Algueró et al., 1988; Brüser et al., 2000
*tcdh*	Thiocyanate dehydrogenase	SCN^−^ → S^0^	Sorokin et al., 2001b
*scnABC*	Thiocyanate hydrolase	SCN^−^ → COS (→H_2_S using COS hydrolase)	Katayama et al., 1998; Arakawa et al., 2007; Ogawa et al., 2013

*^∗^Only dsrABC are shown for clarity. The other genes of this cluster, dsrEFHMKLJOPRS, are present as a single copy in all genomes that contain dsrABC. ^†^Abbreviated as hdr for clarity. The actual operon is hdrC1B1A-hyp-hdrC2B2-lbpA1-dsrE-lbpA2.*

### Distribution of Sulfur Oxidation Genes

#### Sulfide Oxidation: *fcc* and *sqr*

Of the 21 genes/operons shown in Figure 1, five genes—flavocytochrome *c* sulfide dehydrogenase (*fccAB*), *soxAX* and *soxB*—are found in all 75 genomes. Almost half of the strains (37/75) have more than one copy of the flavoprotein subunit *fccB*, although not all have an equal number of corresponding copies of the cytochrome c subunit *fccA*. Strains ALEN2^T^ (*Tv. nitratireducens*) and ARh1^T^ (*Tv. paradoxus*) carry five copies of both genes, whereas strains AL5, ARh4, ALJ4, and ALJ5 have five copies of *fccA*, but only three of *fccB*. Sulfide:quinone oxidoreductase (*sqr*), which also oxidizes sulfide, but donates electrons to the ubiquinone pool rather than to the cytochrome *c* pool, is only found in two strains: ARhD1^T^ (*Tv. thiocyanodenitrificans*) and ALEN2^T^ (*Tv. nitratireducens*), both of which can grow anaerobically by using nitrate as an electron acceptor.

#### Thiosulfate Oxidation: *soxABXYZ*

Genes for *soxAX* and *soxB*, two components of the truncated sox system are present in all genomes included in the study: *soxAX* forms the cytochrome complex that extracts two electrons from the sulfane atom of thiosulfate and binds it covalently to carrier complex *soxYZ*; thiosulfohydrolase *soxB* then catalyzes the hydrolysis of the sulfone group, releasing sulfate. The gene for *soxY* was not found in two genomes of closely related strains *Tv. sulfidiphilus* ALJ17 and *Tv. denitrificans* ALJD^T^ (the latter strain also appears to lack the gene for *soxZ*). The absence of annotated *soxY* in *Thioalkalivibrio* sp. ALJ17 and of *soxYZ* in *Tv. denitrificans* ALJD^T^ appears to be a result of breaks in the genome assemblies. The *soxZ* gene is found on the edge of contig 10 in strain ALJ17 and fragments of *soxY* (annotated as pseudogene features by NCBI) are found on the edges of contigs 5 (locus tag D580_RS0103285) and 54 (D580_RS0114455). Additionally, on contig 54, the partial *soxY* gene is followed by genes for a sigma 54-dependent Fis family transcriptional regulator (D580_RS0114460), a sensor histidine kinase (D580_RS0114465), a small hypothetical protein (D580_RS16435) and a diaminopimelate decarboxylase (D580_RS0114475), all of which are located downstream from *soxY* in *Tv. sulfidiphilus* HL-EbGr7^T^ (Tgr7_0038-0041). In *Tv. denitrificans* the latter three genes are found on the edge of contig 67 (B1C78_RS11375-385).

#### Sulfur Oxidation to Sulfite: *hdr* and *dsr*

The next most abundant set of genes encodes for three subunits of a bacterial heterodisulfide reductase-like system. This is represented in Figure 1 as *hdrABC* for clarity, but the conserved operon is actually *hdrC1B1A-hyp-C2B2*. This operon is closely associated with two lipoate-binding proteins LbpA1 and LbpA2 (Cao et al., 2018), which are found adjacent to the *hdr*-like operon, in all *Thioalkalivibrio* genomes that contain it, as *lbpA1-dsrE-lbpA2*. Only two genomes in the dataset appear to not contain these genes: ARh1^T^ and ARhD1^T^, both of which encode the complete reverse dissimilatory sulfite reductase pathway (*dsrABCEFHMKJOPRS*). In total, six strains in the genus have the rDsr system, which *hdr* is speculated to replace (Quatrini et al., 2009; Cao et al., 2018; Koch and Dahl, 2018): ARh1^T^, ARhD1^T^, ALEN2^T^, ALJ17, HL-EbGr17^T^ (*Tv. sulfidiphilus*) and ALJD^T^. The latter four genomes contain both *dsr* and *hdr*.

#### Sulfite Oxidation to Sulfate: *apr*, *sat*, *sor*, and *soe*

There are three known pathways for the oxidation of sulfite to sulfate in SOB: one, the *aprBA*/*sat* pathway, is indirect and uses adenosine 5′-phosphosulfate as an intermediate compound (Kappler and Dahl, 2001). This pathway was detected in 47 of the 75 genomes, with one additional strain (ALSr1) containing *sat*, but not *aprBA*. The two direct pathways are catalyzed by sulfite:cytochrome *c* oxidoreductase (*sorAB*) or sulfite:quinone oxidoreductase (*soeABC*), although there currently is only genetic evidence for the activity of *soeABC* (Dahl et al., 2013). Putative *soeABC* genes are present in all analyzed strains of *Thioalkalivibrio* and form an operon in all genomes. Genes putatively encoding *sorAB* are present in 60 of the 75 *Thioalkalivibrio* strains.

#### Primary Thiocyanate Degradation: Thiocyanate Dehydrogenase and Thiocyanate Hydrolase

Thiocyanate dehydrogenase (*tcdh*) was found in ten strains in two different genomic contexts, as described in detail previously (Berben et al., 2017). Thiocyanate hydrolase (*scnABC*) was found only in a single strain, ARhD1^T^, which is capable of oxidizing thiocyanate anaerobically.

#### Sulfur Disproportionation: *SOR*

Sulfur oxygenase reductase (*SOR*) was only present in three strains: ARh1^T^, ALEN2^T^, and ALMg11, as was recently described by Rühl et al. (2017).

### Correlation of the Sulfur Oxidation-Related Gene Distribution and the Phylogenetic Structure of the Genus

The dendrogram in Figure 1 represents a hierarchical clustering of *Thioalkalivibrio* genomes based on the presence/absence of sulfur metabolism genes. From left to right, group I, consisting of six strains spanning from ALJ17 (*Tv. sulfidiphilus*) to ARh1^T^ (*Tv. paradoxus*) is characterized by the presence of the rDSR pathway. Additionally, the only occurrences of SQR are within this group (ARhD1^T^ and ALEN2^T^), as well as the only occurrence of thiocyanate hydrolase (ARhD1^T^). Four of these six genomes contain genes for both dissimilatory sulfite reductase and heterodisulfide reductase. ALEN2^T^ and ARh1^T^ are two of the three strains that have sulfur oxygenase-reductase and fall within this group. Three strains—ALJD^T^ (*Tv. denitrificans*), HL-EbGr7^T^ (*Tv. sulfidiphilus*) and ALJ17—have four copies of *soxAX*, with ALJD^T^ containing a fifth *soxX* copy. ALEN2^T^ and ARh1^T^ are positive for the thiocyanate dehydrogenase gene and ARh1^T^ is the only strain in this group lacking a putative *sorAB*. Group II, from ARh5 to AL5, is characterized by the presence of the gene for thiocyanate dehydrogenase, two copies of *soeABC* and *soxB* and the presence of *aprBA* and putative *sorAB* genes. Copy numbers for *fccAB* and *soxAX* are variable within this group. Group III is similar to group II, aside from the lack of TcDH in these strains. Group IV is characterized by the absence of *sorAB* genes. Genes for *aprBA* and *sat* are similarly not found in these genomes, with the exception of strains ALJ3 and ALSr1, the latter of which is the only *Thioalkalivibrio* genome to contain *sat* but not *aprBA*. Genes for *fccAB* and *soeABC* are present in single copy, except in strain ALJ16; *soxX* is present in two copies and *soxA* in one, except in strain ALJ16 which contains an extra copy of both. Group V is characterized by the presence of *sorAB* and *soeABC* and absence of *aprBA*/*sat*. ALMg11 is the only strain outside group I that contains sulfur oxygenase/reductase (SOR), placing it on a separate branch within this group.

The genomic groups row in Figure 1 assigns colors to strains based on their assignment to genomic groups by ANIb analysis, as reported by Ahn et al. (2017). Genomic groups that comprise only a single strain were not assigned a color in this figure to maintain clarity. These labels show that strains belonging to the same genomic groups tend to group together closely based on sulfur gene repertoire. The exceptions to this trend are genomic groups 1 (*Tv. versutus*), as ALMg13-2 is placed in group IV due to its lack of *sorAB*; genomic group 15, due to the placement of strain ALJ3 in group IV rather than group III for the same reason; and genomic group 11 (*Tv. nitratis*), in which the presence of thiocyanate dehydrogenase in strains AL5, ALJ4, ALJ5, and ARh4 leads to their assignment to group II, rather than group III.

### Phylogenetic Analysis of Sulfur Genes

**FIGURE 2:**
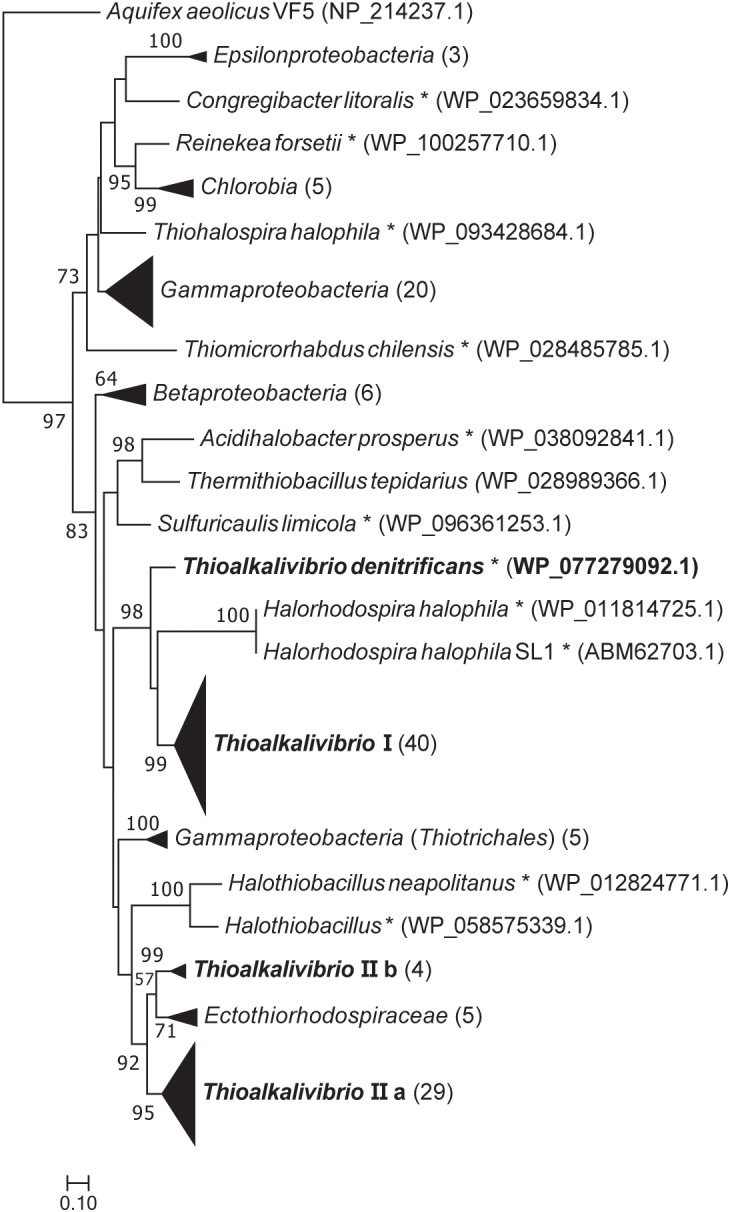
Maximum likelihood tree of SoxB amino acid sequences, based on 250 bootstrap replicates. A group of sequences of 5′-nucleotidase proteins was used as the outgroup, but was pruned from the tree. Sequences of members of the *Gammaproteobacteria* located on branches that could not be further collapsed are marked with an asterisk. Parenthesized numbers indicate the number of sequences contained in a collapsed branch. The scale bar represents % difference.

Figure 2 shows a maximum likelihood phylogenetic tree based on SoxB protein sequences, which is a unique marker of the Sox pathway (Meyer et al., 2007). All *Thioalkalivibrio* sequences were included, as well as the SoxB sequences of forty other SOB species in a variety of phylogenetic classes. Whereas most classes cluster together, the gammaproteobacterial sequences are found distributed across the tree. One cluster of *Gammaproteobacteria* is split by the anoxygenic green sulfur bacteria (class *Chlorobia*) and the *Epsilonproteobacteria*. A second group of gammaproteobacterial SoxB sequences forms a sister taxa to SoxB sequences of the *Betaproteobacteria*. The only non-gammaproteobacterial sequence in this cluster belongs to *Thermithiobacillus tepidarius*. Sequences from *Thioalkalivibrio* are found in four distinct groups: group I contains a single SoxB sequence from every *Thioalkalivibrio* strain, with the exception of *Tv. nitratireducens* (ALEN2^T^), *Tv. paradoxus* (ARh1^T^) and the phylogenetically divergent strains ARhD1 (*Tv. thiocyanodenitrificans*), HL-EbGr7^T^ and ALJ17 (*Tv. sulfidiphilus*). *Tv. denitrificans* (ALJD^T^) is split from group I by SoxB sequences from *Halorhodospira halophilus*. Group IIa contains all *Thioalkalivibrio* strains which possess two copies of the *soxB* gene, as well as strains ALEN2^T^ and ARh1^T^. Group IIb contains sequences of strains ARhD1^T^, HL-EbGr7^T^, ALJ17, and ALJD^T^, and is separated from group IIa by a group of sequences belonging to *Ectothiorhodospira*, *Ectothiorhodosinus* and one strain of *Halorhodospira*.

Trees based on amino acid sequences for SoxY and SoxZ show similar phylogenetic distributions (see Supplementary Figures S1, S2), however, the bootstrap values in these trees are much lower.

**FIGURE 3:**
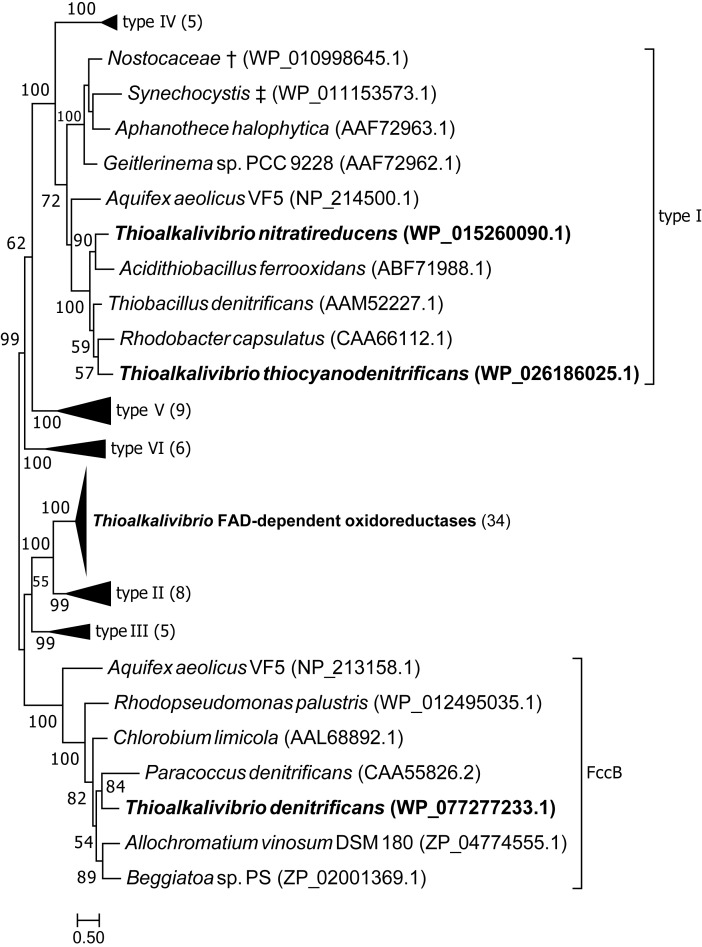
Maximum likelihood tree of SQR amino acid sequences, based on a previously published tree classifying SQR into six groups (Marcia et al., 2010). Sequences from ortholog group 1954 were placed in the tree, as well as sequences similar to SQR by blastp searches of the genomes of strains ALJD^T^, ARhD1^T^ and ALEN2^T^. ^†^ Multispecies record representing *Nostoc* sp. PCC 7120 and *Trichormus variabilis* NIES-23; ^‡^ multispecies record representing *Synechocystis* sp. PCC 6803 and *Synechocystis* sp. IPPAS B-1465. Parenthesized numbers indicate the number of sequences contained in a collapsed branch. The scale bar represents % difference.

Figure 3 shows the position of putative SQR sequences from *Thioalkalivibrio* in a previously published phylogenetic tree, where structure information was used to define six types of SQR (Marcia et al., 2010). Candidate SQR sequences from *Thioalkalivibrio* were assigned to a single ortholog group by the orthoMCL algorithm. Similar sequences from *Tv. denitrificans* ALJD^T^, *Tv. nitratireducens* ALEN2^T^ and *Tv. thiocyanodenitrificans* ARhD 1^T^ were identified by blastp and added to this group and used to generate a new SQR tree together with the sequences used by Marcia et al. (2010). The figure clearly shows that the putative SQR sequences from ALEN2^T^ and ARhD1^T^ fall within group I, whereas the sequence from ALJD^T^ is a flavocytochrome *c* sulfide dehydrogenase FccB [which is confirmed by the presence of a gene coding for a diheme cytochrome *c* subunit (FccA) adjacent to it, locus tag B1C78_RS00715]. The FAD-dependent oxidoreductase sequences contained in ortholog group 1954 form a sister taxa to type II SQR. A recent reclassification of SQR and sulfide dehydrogenases provided by Sousa et al. (2018) maintains consistency with the previously defined type II SQRs (renamed group C), whereas type I SQRs form group D together with types V and VI.

**FIGURE 4:**
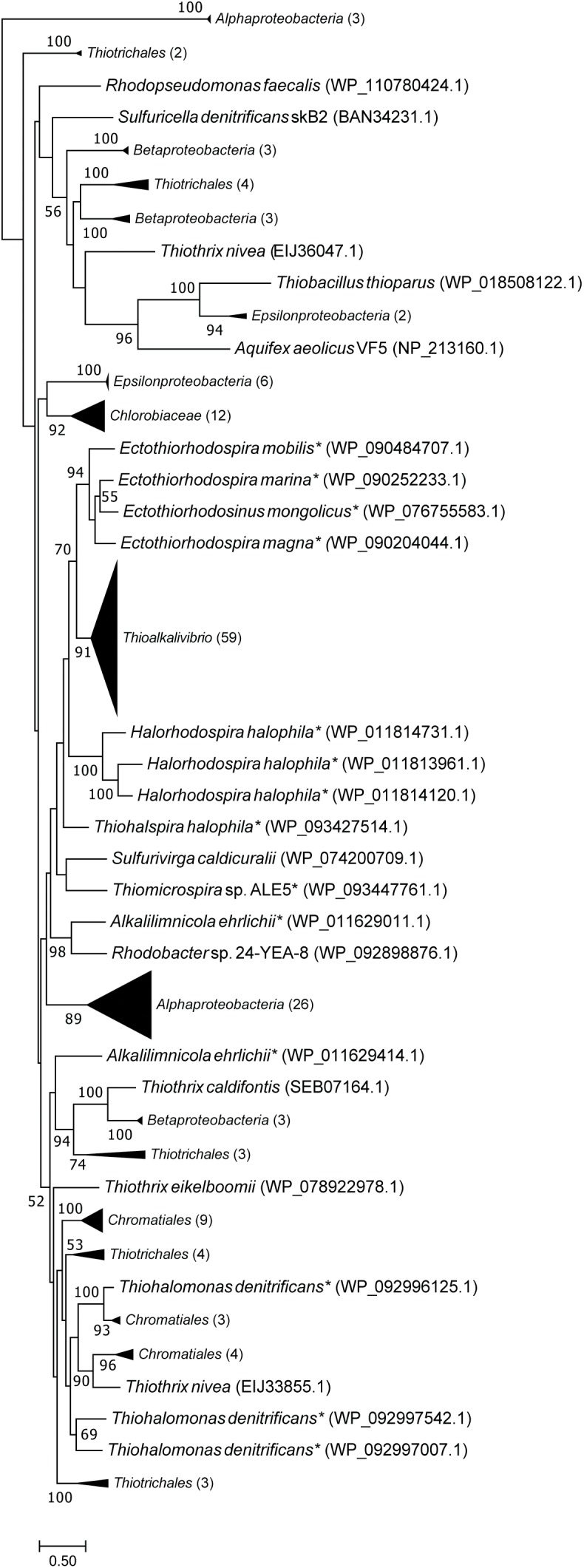
Maximum likelihood tree of FccB (the flavoprotein subunit of flavocytochrome *c* sulfide dehydrogenase) sequences, based on 500 bootstrap replicates. Members of the family *Ectothiorhodospiraceae* have been marked with an asterisk. This tree includes all FccB copies shown in Figure 1, identical sequences have been collapsed by NCBI into multispecies records, leaving 59 unique sequences. Parenthesized numbers indicate the number of sequences contained in a collapsed branch. The scale bar represents % difference.

Figure 4 is a maximum likelihood tree of amino acid sequences of FccB, the catalytic subunit of sulfide dehydrogenase. In contrast to SoxB, all FccB sequences from *Thioalkalivibrio* are located in a single cluster. The most closely related sequences are from the other members of the *Ectothiorhodospiraceae*, which mostly cluster together. The exceptions to this are one sequence of *Alkalilimnicola erlichii*, which is found as a sister branch to a group of sequences from the *Thiotrichales* and *Betaproteobacteria*, and *Thiohalomonas denitrificans* (*Gammaproteobacteria*), whose three FccB sequences are located on a branch with mostly other *Chromatiales* sequences and a single *Thiothrix nivea* sequence, followed by the *Alphaproteobacteria*. Sequences from the *Betaproteobacteria* and gammaproteobacterial orders *Chromatiales* and *Thiotrichales* are scattered throughout the tree.

The putative SoeA sequences from *Thioalkalivibrio* strains form two groups in a tree (Supplementary Figure S3) which includes previously predicted SoeA amino acid sequences from other SOB according to Dahl et al. (2013). Group *Thioalkalivibrio* I contains a representative sequence from every strain, although the phylogenetically divergent strains ALJD^T^, ARhD1^T^, HL-EbGr7^T^, and ALJ17 are separated from this group with 100% bootstrap support. *Thioalkalivibrio* group II contains all other SoeA sequences present in the genomes studied here and appears to be more distantly related to the other sequences in the tree, although bootstrap support for this branch is low. The putative SoeA sequences in group II have an average sequence similarity of 55% with those in group I.

### Genomic Context of Genes Present in Multiple Copies

**FIGURE 5:**
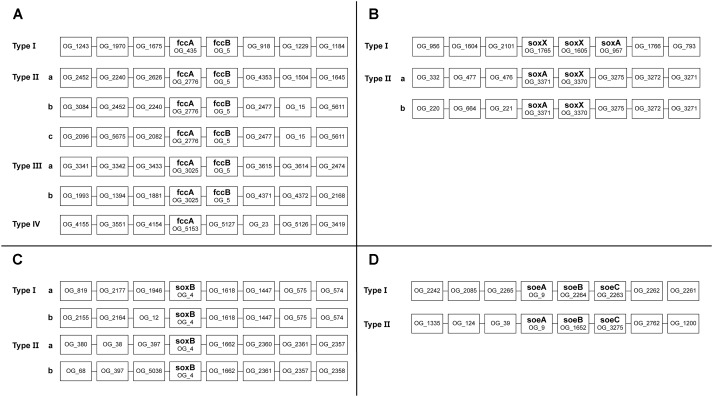
Schematic representation of the genomic contexts of genes present in multiple copies in *Thioalkalivibrio* genomes. Selected genes were *fccA* **(A)**; *soxX* **(B)**; *soxB* **(C)**, and *soeA* **(D)**.

To assess the possible function of multiple copies of the same gene present in *Thioalkalivibrio* genomes, an overview of genomic contexts was generated. Figure 5 shows a schematic representation of this data. Genomic contexts for *hdrBC* were not included as they are usually present in a single operon (Koch and Dahl, 2018); contexts for *dsrAB* are not shown as only six genomes contain *dsr* genes at all, and only two of these contain multiple copies.

Sequences for *fccA* were assigned to four different ortholog groups by OrthoMCL (Figure 5A). Group OG_435 contains a sequence from every *Thioalkalivibrio* strain and upstream and downstream contexts are highly similar in all genomes. Strain ALJ16 is an exception to this, as it contains two OG_435 *fccA* sequences, one of which does not conform to this context. OG_2776 and OG_3025 *fccA* genes are also always found adjacent to *fccB*, but show more divergent upstream and/or downstream contexts (see Figure 5A). The *fccA* sequences that are present in the TcDH operon (Berben et al., 2017) are found in this ortholog group, and only these have the type IIIa context. Only a single *fccA* sequence was assigned to ortholog group OG_5153, from strain AKL11, and it is not found in an operon with *fccB*.

A majority of strains have two copies of *soxX* and a single copy of *soxA* (Figure 1). *SoxX* sequences were assigned to three different ortholog groups: OG_1765 and OG_1605 sequences were found directly adjacent to each other, with *soxA* directly downstream (Figure 5B). *SoxX* sequences assigned to OG_3370 all have a gene for *soxA* directly adjacent (Figure 5B). *Tv. sulfidiphilus* (HL-EbGr7^T^ and ALJ17^T^) and *Tv. denitrificans* (ALJD^T^) contain four repeats of *soxXA* in a row, with an additional *soxX* copy in ALJD^T^. All *soxB* sequences were assigned to a single ortholog group (OG_4). There are two major genotypes, as shown in Figure 5C. Type I comprises a *soxB* sequence from every *Thioalkalivibrio* genome studied, whereas Type II contains a sequence from every strain with two *soxB* copies. Type I is divided into subtypes by 12 strains which have a divergent genomic context (Figure 5C).

A total of 42 strains have two copies of *soeABC* (Figure 1), of which all *soeA* sequences are found in a single ortholog group. These sequences are found in two genomic contexts: all strains have a *soeABC* cluster in a type I context; additional *soeABC* copies are found in context type II (Figure 5D).

## Discussion

### Only Six Strains Have a Fully Characterized Sulfur Oxidation Pathway

With the information that is currently available, a complete sulfur oxidation pathway (from sulfide to sulfate) can only be reconstructed for six *Thioalkalivibrio* strains: ARhD1^T^ (*Tv. thiocyanodenitrificans*), ALJD^T^ (*Thioalkalivibrio denitrificans*), HL-EbGr7^T^ (*Tv. sulfidiphilus*), ALJ17, ARh1^T^ (*Tv. paradoxus*), and ALEN2^T^ (*Tv. nitratireducens*). These six strains are capable of zero-valent sulfur oxidation using the reverse dissimilatory sulfite pathway, which was originally characterized in the purple sulfur bacterium *Allochromatium vinosum* (Frigaard and Dahl, 2009). These strains were previously shown to be phylogenetically divergent from the other *Thioalkalivibrio* strains, both by 16S rRNA gene analysis and modern phylogenomic methods (Ahn et al., 2017), and may need to be assigned to two different genera in the future. No components of the *dsrABEFHCMKLJOPNRS* cluster were detected in any of the remaining strains, although *aprBA* and *sat*, required for the activation of sulfate in sulfate-reducing bacteria (SRB), were detected in 47 genomes. The oxidation of sulfur in the strains lacking rDsr is more extensively discussed below.

The detection of partial *soxYZ* genes on contig edges in the genomes of ALJD^T^ and ALJ17, together with the fact that both strains are routinely cultured with thiosulfate as the sole electron donor, support the conclusion that these strains do indeed carry the *soxYZ* genes. Conclusive proof, however, can only be obtained by either performing additional sequencing runs to close the genome, or by PCR with primers designed using *soxYZ* sequences of closely related strains. The inclusion of these *soxY* in ALJ17 and *soxYZ* in ALJD^T^ does not change the order of the dendrogram in Figure 1, only the branch lengths.

### Clustering of Strains Based on Sulfur Metabolism Mirrors Classification of Genomic Groups Based on ANIb Analysis

Hierarchical clustering of *Thioalkalivibrio* strains based on the presence/absence of sulfur oxidation-related genes leads to a dendrogram in which strains that are assigned to individual genomic groups are found on the same branch. Whereas the sulfur gene clustering broadly groups strains within their genomic groups, the order of these clusters does not mirror the phylogenomic distribution of strains. For example, in ANIb analysis, the Kenyan isolate ALJT was shown to be most closely related to another Kenyan strain ALJ16, but based on sulfur gene content it is more similar to strain ALMg11 and the *Tv. versutus*/*Tv. jannaschii* group. Similarly, the location in which the strain was initially found seems to be uncorrelated to the distribution of sulfur genes in its genome. Ultimately, the major differences between *Thioalkalivibrio* strains are the presence or absence of *aprBA*/*sat*, *sorAB*, and the presence of thiocyanate dehydrogenase in ten genomes. The *dsr* genes, *sqr* and sulfur oxygenase/reductase are only found in strains that are phylogenetically divergent from the rest of the genus (with the exception of SOR in ALMg11) and thiocyanate hydrolase is only found in a single species, *Tv. thiocyanodenitrificans*.

### Phylogenetic Distribution Based on SoxB Sequences Mirrors That of the 16S rRNA Gene Sequences

The maximum likelihood tree of SoxB amino acid sequences follows a pattern previously seen in the 16S rRNA gene sequence analyses of the type strains of *Thioalkalivibrio* (Sorokin et al., 2013). Herein, type strains ARhD1^T^ (*Tv. thiocyanodenitrificans*), ALJD^T^ (*Tv. denitrificans*) and HL-EbGr7^T^ (*Tv. sulfidiphilus*) are separated from the remaining type strains by the anoxygenic phototrophic SOB of the *Ectothiorhodospiraceae* family, such as *Ectothiorhodosinus*, *Ectothiorhodospira*, and *Thiorhodospira*. This holds true for both group I and group II in the SoxB tree (Figure 2), although in group I strain ALJD^T^ is split off by *Halorhodospira* sequences rather than by *Ectothiorhodospira* and *Ectothiorhodosinus* sequences. The division between the ‘core’ *soxB* gene (groups IIa and IIb combined contain a single representative from all strains) and the ‘accessory’ *soxB* gene (group I comprises a sequence from all strains that have two copies of *soxB*, except strain ALJD^T^) is interesting. A second copy of *soxB* is present in all strains comprising the clade of species 1–15 in the ANIb tree of *Thioalkalivibrio* (Ahn et al., 2017; Figure 2). This is possibly the result of a single duplication/horizontal gene transfer event, although it is not possible to infer this definitively from the available data. In this hypothesis, strain ALJD^T^ acquired its second copy of *soxB* through a separate event. A phylogenetic tree based on AprBA amino acid sequences published previously by Watanabe et al. (2016; Supplementary Figure S3, therein) similarly shows a separation of *Tv. thiocyanodenitrificans* and *Tv. sulfidiphilus* from the rest of the *Thioalkalivibrio* sequences (this tree does not contain sequences from *Tv. denitrificans* and strain ALJ17), although in this case they are found on a branch with sequences from the family *Chromatiaceae*.

The phylogenetic tree of FccB (Figure 4), the catalytic FAD-containing subunit of flavocytochrome c, shows all *Thioalkalivibrio* sequences on a single branch, closely related to other *Ectothiorhodospiraceae* sequences. On the other hand, sequences from other *Chromatiales* and *Thiotrichales* SOB, as well as the betaproteobacterial SOB, are found in multiple clusters spread across the tree, which may be the result of horizontal gene transfer events. However, bootstrap values for most branches are low, making it impossible to infer a strong conclusion from this data.

### Genomic Contexts of Multi-Copy Genes

Of the four ortholog groups that contain *fccA* sequences, only one contains a representative from all *Thioalkalivibrio* strains. One of the *fccA* copies found in strain AKL11 was not located next to an *fccB* gene and may not function as the electron acceptor to sulfide dehydrogenase. The remaining *fccA* ortholog groups show a diversity of genomic contexts and we hypothesize that these *fccAB* clusters were acquired through horizontal gene transfer. The *in vivo* role of multiple *fccAB* genes will have to be confirmed with physiological experiments. Likewise, it is not known whether the presence of different genes in the direct genomic context of *fccAB* has any influence on its expression. A comparison with genome contexts of *fccAB* in a diverse group of sulfur-oxidizing bacteria using IMG (Markowitz et al., 2014), including *Allochromatium vinosum*, *Chlorobium limicola*, *Paracoccus denitrificans*, and *Thiobacillus denitrificans* showed no conservation of surrounding genes.

The function of additional copies of *soxX* is even less clear, as these are in most cases found in *soxX*-*soxX*-*soxA* operons and three of the phylogenetically divergent strains (ALJD^T^, HL-EbGr7^T^, and ALJ17) have *soxX*-*soxA*-*soxX*-*soxA*-*soxX*-*soxA*-*soxX*-*soxA* operons. In this case, genetic knock-out experiments would likely provide insight into the function of these genes; however, no generically applicable system for genetic manipulation of *Thioalkalivibrio* currently exists. Additionally, gene expression studies could be used to study the relative expression these multiple copies and show whether all, or just some, are utilized during thiosulfate oxidation.

Genomic contexts for *soxB* and *soeA* show a mostly straightforward picture: one genotype which is present in all *Thioalkalivibrio* strains and one which is present in genomes that have additional copies of these genes. Phylogenetic analysis of *soxB* showed that the second copies of this gene present in 57 genomes were likely acquired from related *Ectothiorhodospiraceae* (Figure 2).

### Common Pathways for Sulfur Oxidation

A number of sulfur oxidation genes/pathways shown in Figure 1 were detected in each of the 75 strains analyzed in this study. These are: *fccAB*, which catalyzes the oxidation of sulfide; the truncated *sox* system, *soxAXYZB*, which oxidizes thiosulfate to sulfur and sulfate; and *soeABC*, which oxidizes sulfite to sulfate. A major open question is the pathway involved in the oxidation of sulfur to sulfite. This separates the *Thioalkalivibrio* strains analyzed in this study in two groups: six strains in which the reverse dissimilatory sulfite pathway was detected and 69 strains which were rDsr-negative. The taxonomic status of four of the six rDsr-positive *Thioalkalivibrio* strains has been questioned, whereas phylogenomic analysis of *Tv. paradoxus* ARh1^T^ and *Tv. nitratireducens* ALEN2^T^ was ultimately inconclusive (Ahn et al., 2017).

A sulfide oxidation mechanism that involves the enzyme persulfide dioxygenase (PDO), which oxidizes the persulfide group of glutathione persulfide (GSSH) to produce GSH and sulfite, was described for heterotrophic sulfur-oxidizing bacteria (Xin et al., 2016; Xia et al., 2017). However, this pathway involves sulfide:quinone reductase, which was only detected in two *Thioalkalivibrio* strains—both of which are rDsr-positive. Additionally, transcriptomics experiments with persulfide dioxygenase knock-out mutants of *Acidithiobacillus caldus* did not support a role for PDO in sulfur oxidation in this rDsr and SoxCD negative species (Wu et al., 2017). Another candidate pathway for the oxidation of sulfur is the Hdr-like proteins, which were detected in the genomes of all rDsr-negative strains, as well as in four rDsr-positive ones (ALJD^T^, HL-EbGr7^T^, ALJ17, and ALEN2^T^). Although *in vitro* biochemical evidence that the Hdr-like pathway catalyzes this reaction has not been published to date, transcriptomics experiments have demonstrated its over expression during growth with sulfur compounds in *Acidithiobacillus ferrooxidans* (Quatrini et al., 2009) and a recent study involving the dimethyl sulfide-oxidizing bacterium *Hyphomicrobium denitrificans* showed an increase in Hdr enzyme production during growth on thiosulfate. In the same study it was demonstrated that disrupting the *hdr* operon abolished thiosulfate oxidation, leaving only a low rate of thiosulfate consumption through a different pathway involving the formation of tetrathionate (Koch and Dahl, 2018). Another recent study highlighted the association of the *hdr*-like operon with lipoate-binding proteins, which are proposed to function as sulfur carriers presenting the substrate to the catalytic sites of the Hdr-like complex (Cao et al., 2018).

Another question is why many genomes contain multiple putative pathways for the oxidation of sulfite to sulfate. While putative *soeABC* genes were detected in the genomes of all strains in this study, they are the sole genes implicated in sulfite oxidation in only 12 strains. All other strains contain various combinations of *soeABC*, *aprBA*, and *sorAB*. Additionally, *sorAB* was previously reported as not widely distributed among *Ectothiorhodospiraceae* (Ghosh and Dam, 2009). The *aprBA* genes were shown to not be essential for sulfite oxidation in *Allochromatium vinosum*, although they did confer a growth advantage under specific conditions (Dahl, 1996; Sánchez et al., 2001). Since complete oxidation of reduced sulfur compounds to sulfate is a common trait among *Thioalkalivibrio* species, we had expected to find at least one sulfite oxidation system to be ubiquitous among the strains studied here. The only candidate for this currently is *soeABC*, but phylogenetic analysis shows that not all sequences group together in a tree, which would be expected for a core metabolism gene. The role and importance of *soeABC*, *sorAB*, and *aprBA* in sulfite oxidation in *Thioalkalivibrio* will have to be studied in more detail using gene expression studies and genetic manipulation.

**FIGURE 6:**
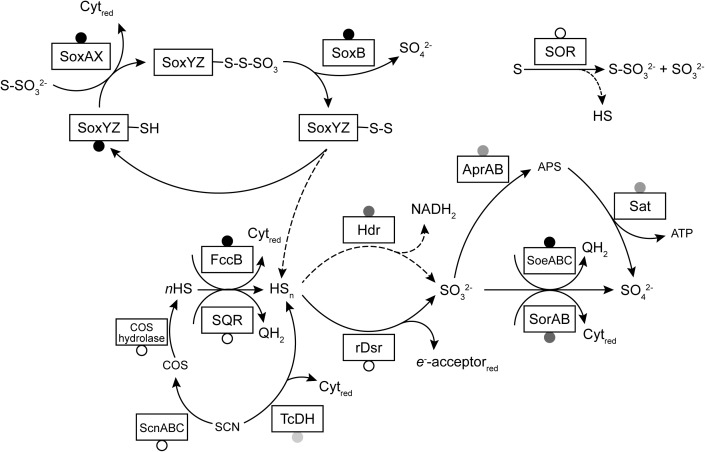
A model of sulfur oxidation pathways in *Thioalkalivibrio*. Boxes represent enzymes for which genes were detected in the studied genomes. Circles indicate the prevalence of these genes: black, present in all genomes; gray, present in >10 genomes (and <75); unfilled, present in <10 genomes. Dashed lines indicate reactions with unknown mechanism (in the case of sulfane sulfur dissociation from SoxYZ) or low rates (sulfur reduction to sulfide by SOR). The reactions of rDsr and the Hdr-like system have been simplified for clarity.

A model of sulfur metabolism in *Thioalkalivibrio* is shown in Figure 6, similar to earlier models based on a single strain (Ang et al., 2017) or a limited set of thiocyanate-utilizing species (Berben et al., 2017).

### Rare Sulfur-Oxidation Related Genes

Apart from the genes of the rDsr pathway, as discussed above, there are four other genes that are uncommon among *Thioalkalivibrio*. The distribution and genomic context of thiocyanate dehydrogenase (*tcdh*; found in 10 genomes) and thiocyanate hydrolase (*scnABC*; found in a single genome) were discussed in detail in a previous study (Berben et al., 2017), where it was shown that the *tcdh* gene is found in two genomic contexts (one in ARh 1^T^ and ALEN 2^T^ and one in the other cyanate-pathway using strains) that include genes for *fccAB*, copper-resistance proteins (*copCD*) and a twin-arginine transporter (*tatA*). Sulfur oxygenase/reductase (SOR) from *Tv. paradoxus* was recently biochemically characterized and it was shown that the enzyme has an atypically low reductase activity (Rühl et al., 2017), although the *in vivo* role of SOR in *Thioalkalivibrio* has not been determined yet. Genes for tetrathionate degradation were not detected at all in the genomes of *Thioalkalivibrio*, which appears to contradict reported tetrathionate utilization by some species. However, tetrathionate is unstable in alkaline environments and can spontaneously decompose to form thiosulfate, when the pH is greater than 9 (Varga and Horváth, 2007). Carbon disulfide utilization, at low rates, was only reported for *Tv. paradoxus* ARh 1 (Sorokin et al., 2002b) and the biochemistry of this reaction remains unknown. A putative homolog of carbon disulfide hydrolase (Smeulders et al., 2011) was not detected in this genome. While oxygen-dependent CS_2_ consumption was demonstrated in cell-free extracts of *Paracoccus denitrificans* (Jordan et al., 1997), no further enzymatic characterization was performed. The genome of *Tv. nitratireducens* ALEN 2^T^ is the only one to contain a gene encoding a putative carbon disulfide hydrolase, but possible growth with CS_2_ was never tested for this strain.

The phylogenetic tree of putative SQR sequences shown in Figure 3 shows that sequences from *Tv. nitratireducens* ALEN2^T^ and *Tv. thiocyanodenitrificans* ARhD1^T^ fall within the type I SQR, as defined by Marcia et al. (2010). The SQR enzymes of this group were shown to have a high affinity for sulfide and are involved in respiratory processes, as well as in photosynthesis in phototrophic sulfide oxidizers (Marcia et al., 2010). In ALEN2^T^ and ARhD1^T^ SQR is likely required for anaerobic growth on sulfide, although this does not explain the fact that SQR was not detected in the genome of *Tv. denitrificans* ALJD^T^, which is also capable of anaerobic growth (Sorokin et al., 2001a). The remaining *Thioalkalivibrio* sequences form a sister clade to type II SQR that is strongly supported by bootstrap analysis. Type II SQR is present in eukaryotes and bacteria. Although SQR activity has been demonstrated in bacterial type II SQR (Han and Perner, 2016; Shen et al., 2016), the affinity for sulfide is low (in the millimolar range) and their physiological roles are unclear (Marcia et al., 2009, 2010). In *Thioalkalivibrio*, gene expression studies and biochemical characterization of the expressed protein are necessary to determine whether this group is active in sulfide oxidation or has an alternative function.

## Conclusion

In this paper we have presented the diversity and distribution of genes related to sulfur oxidation in 75 strains of *Thioalkalivibrio*, a group of haloalkaliphilic and chemolithoautotrophic SOB from soda lakes. We have shown that flavocytochrome *c*, the truncated *sox* system (*soxAXYZB*) and sulfite:quinone oxidoreductase (*soeABC*) are present in all strains. The pathway from elemental sulfur to sulfite is currently not resolved for all *Thioalkalivibrio*, as only six genomes encode the dissimilatory sulfite reductase system. The *hdr*-like operon is a good candidate for sulfur oxidation, although the release of sulfite from this enzyme system has yet to be demonstrated. It is found in all *dsr*-negative strains and the genomes of four strains contain both, although the physiological consequences of this are unknown.

Hierarchical clustering showed that the sulfur gene repertoire of individual strains correlates well with genomic groups previously defined by ANIb analysis. Phylogenetic analysis of SoxB, FccB, and SoeA amino acid sequences reaffirms the complex evolutionary history of *Thioalkalivibrio* that was reported in previous analyses of 16S rRNA and CbbL sequences (Tourova et al., 2006; Ahn et al., 2017). Genomic contexts of genes present in multiple copies show that there is generally one genotype with sequences from all strains and one or more genotypes additional copies that were likely acquired through HGT.

There are a number of major unresolved questions regarding the sulfur metabolism of *Thioalkalivibrio*, most notably the oxidation of elemental sulfur as mentioned above. The *in vivo* function of the FAD-dependent oxidoreductases that form a sister group to type II SQR sequences provides another avenue for further research.

## Author Contributions

TB, LO, DS, and GM contributed to the study design. LO performed the grouping of annotated genes in ortholog groups. TB analyzed the distribution and phylogeny of specific sulfur metabolism-related genes and drafted the manuscript. LO, DS, and GM provided feedback on the analyses and critically reviewed the manuscript. All authors read and approved the final version of the manuscript.

## Conflict of Interest Statement

The authors declare that the research was conducted in the absence of any commercial or financial relationships that could be construed as a potential conflict of interest.

## Supplementary Material

FIGURE S1 Maximum likelihood tree of SoxY sequences, based on 500 bootstrap replicates. Sequences of desulfoferredoxin-family proteins were used as the outgroup and pruned from the tree. The scale bar represents % difference.

FIGURE S2 Maximum likelihood tree of SoxZ sequences, based on 500 bootstrap replicates. Sequences of quinoprotein dehydrogenase-associated SoxYZ-like proteins were used as outgroup and later pruned from the tree. The scale bar represents % differences.

FIGURE S3 Maximum likelihood tree of SoeA sequences, based on 500 bootstrap replicates. Sequences of sulfite:cytochrome *c* oxidoreductase subunit A (SorA) were used as outgroup and pruned from the tree. The scale bar represents % differences. Bootstrap values below 50% are not shown. Parenthesized numbers indicate the number of sequences included in a collapsed branch.

TABLE S1 Overview of *Thioalkalivibrio* strains included in this study, including geographical location from which the strain was isolated, genome size, number of protein-coding genes, genome accession number and the results of the CheckM analysis.

TABLE S2 Groups of orthologous proteins as predicted by OrthoMCL. Rows represent the different ortholog groups. Accession numbers of protein sequences included in an ortholog group are separated by tabs on the same line.
