# Supplementary figures and images for: Diversity and Distribution of Sulfur Oxidation-Related Genes in Thioalkalivibrio, a Genus of Chemolithoautotrophic and Haloalkaliphilic Sulfur-Oxidizing Bacteria
Source: Front Microbiol. 2019 Feb 14;10:160. doi: 10.3389/fmicb.2019.00160 (PMC6382920; doi:10.3389/fmicb.2019.00160)

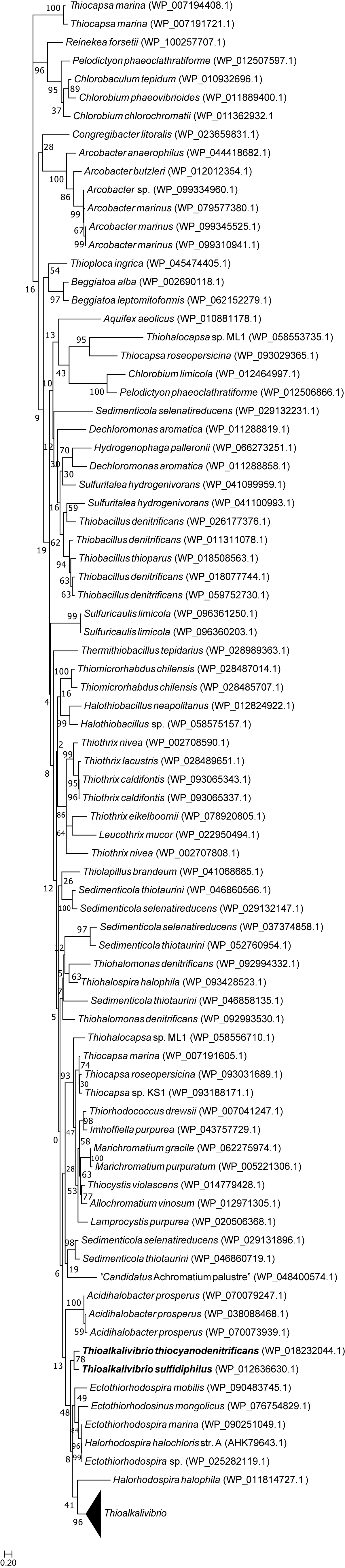

Supplement: FIGURE S1 — Maximum likelihood tree of SoxY sequences, based on 500 bootstrap replicates. Sequences of desulfoferredoxin-family proteins were used as the outgroup and pruned from the tree. The scale bar represents % difference. [file Image_1.TIF]

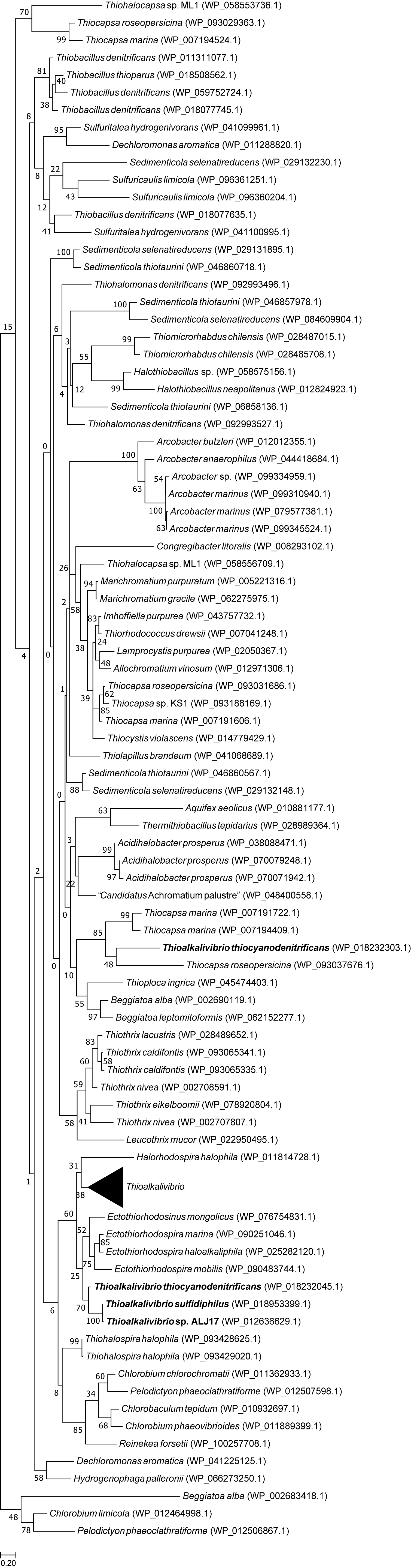

Supplement: FIGURE S2 — Maximum likelihood tree of SoxZ sequences, based on 500 bootstrap replicates. Sequences of quinoprotein dehydrogenase-associated SoxYZ-like proteins were used as outgroup and later pruned from the tree. The scale bar represents % differences. [file Image_2.TIF]

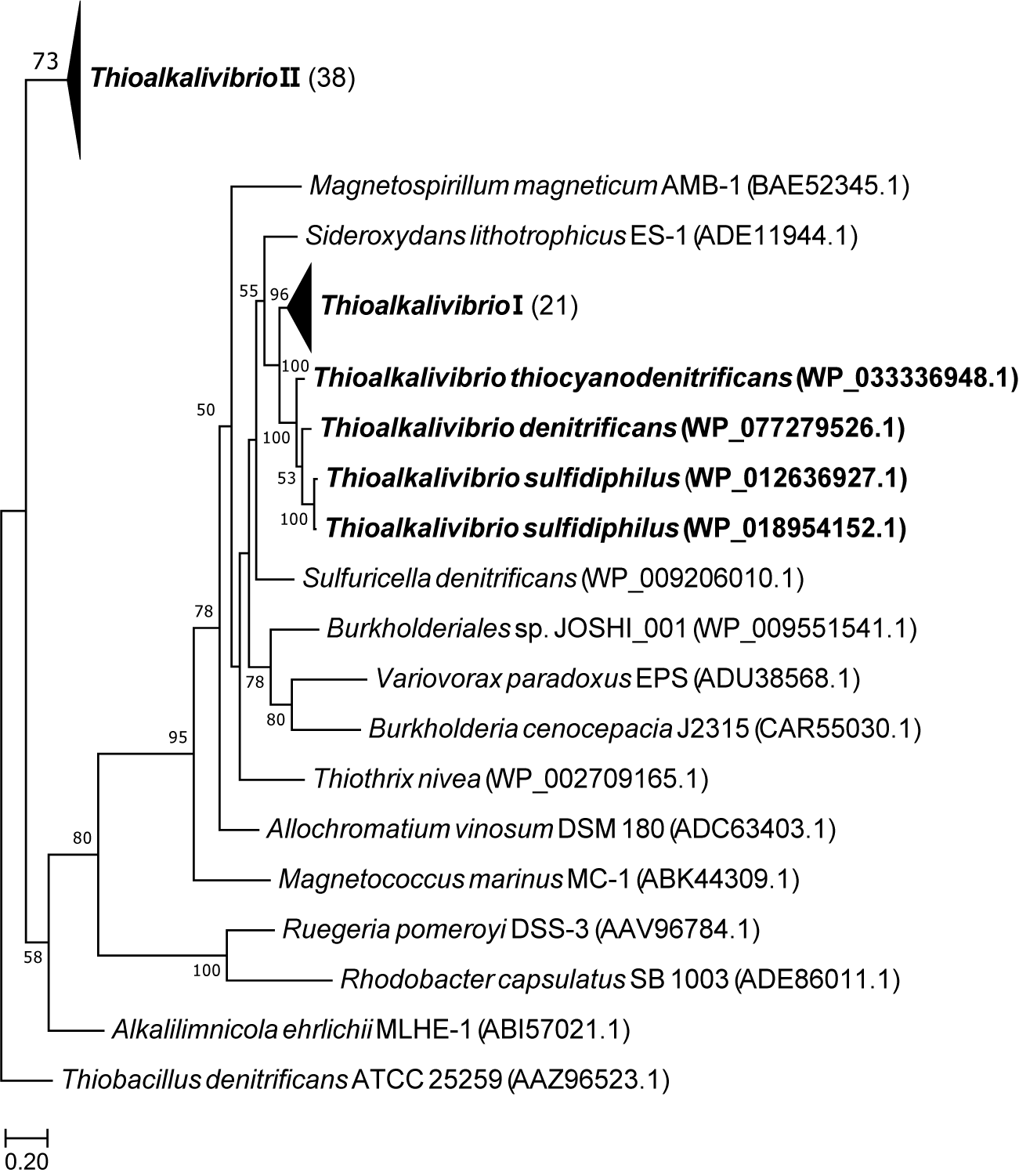

Supplement: FIGURE S3 — Maximum likelihood tree of SoeA sequences, based on 500 bootstrap replicates. Sequences of sulfite:cytochrome c oxidoreductase subunit A (SorA) were used as outgroup and pruned from the tree. The scale bar represents % differences. Bootstrap values below 50% are not shown. Parenthesized numbers indicate the number of sequences included in a collapsed branch. [file Image_3.TIF]
